# Assessment of extremely premature lambs supported by the Extrauterine Environment for Neonatal Development (EXTEND)

**DOI:** 10.1038/s41390-024-03287-0

**Published:** 2024-06-04

**Authors:** Maureen Peers de Nieuwburgh, Apeksha Dave, Sameer A. Khan, Michelle Ngo, Kevin B. Hayes, Matthew Slipenchuk, Evan Lieberman, Mohanad R. Youssef, Dan Crompton, Alia Mohsin Choudhry, Nan Guo, Zhiyun Tian, Jack Rychik, Marcus G. Davey, Alan W. Flake

**Affiliations:** 1 Center for Fetal Research, Children’s Hospital of Philadelphia, Philadelphia, PA USA; 2 Division of Cardiology, Department of Pediatrics, The Children’s Hospital of Philadelphia, Philadelphia, PA USA; 3 Vitara Biomedical Inc., Philadelphia, PA USA; 4 Department of Radiology, Hospital of the University of Pennsylvania, Philadelphia, PA USA

## Abstract

**Background:**

Our team has previously reported physiologic support by the EXTra-uterine Environment for Neonatal Development (EXTEND) of 105 to 117 days gestational age (GA) lambs for up to 28 days with normal organ maturation. However, the fetal lamb brain matures more rapidly, requiring the study of 90-105 day GA fetal lambs to assess more neurodevelopmentally equivalent lambs to the 23–25 week GA extreme premature infant.

**Methods:**

Extremely preterm lambs (90–95 days of GA) were delivered by C-section and supported by EXTEND. Estimated circuit flows were maintained at around 325 ml/kg/min. After support on EXTEND, MRI and histopathologic analysis were performed and compared to 105–112 days GA control lambs.

**Results:**

The extremely preterm group includes 10 animals with a mean GA of 91.6 days, a mean weight at cannulation of 0.98 kg and a mean length of stay on EXTEND of 13.5 days (10–21 days). Hemodynamics and oxygenation showed stable parameters. Animals showed growth and physiologic cardiac function. MRI volumetric and diffusion analysis was comparable to controls. Histologic brain analysis revealed no difference between study groups.

**Conclusion:**

EXTEND appears to support brain and cardiac development in an earlier gestation, less mature, lamb model.

**Impact:**

- Prolonged (up to 21 days) physiological support of extremely preterm lambs of closer neurodevelopmental equivalence to the 24–28 gestational week human was achieved using the EXTEND system.
- EXTEND treatment supported brain growth and development in extremely preterm fetal lambs and was not associated with intraventricular hemorrhage or white matter injury.
- Daily echocardiography demonstrated physiologic heart function, absence of cardiac afterload, and normal developmental increase in cardiac chamber dimensions.
- This study demonstrates hemodynamic and metabolic support by the EXTEND system in the extremely preterm ovine model.

## Introduction

Despite incremental improvements in survival and care of extreme premature infants, there has been no corresponding reduction in major morbidity. There are now more patients who suffer from the sequelae of prematurity than a decade ago.^1–3^ The extreme premature infant (22–28 weeks of GA) is developmentally immature, and current technology applied to their care is non-physiologic and associated with significant iatrogenic injury.^3,4^ Among these, neurologic injury is one of the most devastating with high rates of severe neurodevelopmental disabilities from birth to adulthood.^5^ Patel et al.^6^ reported that 9% of deaths in extremely premature infants are caused by primary central nervous system (CNS) injury and 18% of deaths due to other complications have concomitant CNS injury. Furthermore, long term sequela of prematurity includes serious neurodevelopmental disabilities such as cerebral palsy (with incidence ranging from 1% at 34 weeks to 20% at ≤ 26 weeks of gestation), intellectual and psychological impairment, blindness, hearing loss, and epilepsy.^7–11^ Therefore, there is a need to develop new, more physiologic strategies for managing these patients that enable ongoing organ growth and maturation.

Our laboratory previously reported on the development of an extrauterine device that provided physiological support to preterm fetal lambs for up to four weeks with a successful transition to postnatal life.^12^ The EXTra-uterine Environment for Neonatal Development (EXTEND) system maintains normal fetal physiology, circulation, and organ development. With appropriate nutrition, lambs supported by the EXTEND system demonstrated normal growth and maturation^13,14^ with a subset of lambs successfully transitioning to postnatal life.^12^ In our earlier studies,^12^ a 105–111 gestation day model (1.0–1.5 kg) was utilized to target pulmonary developmental equivalence (canalicular stage) to the human extreme preterm human at 23–25 weeks of gestation. However, the 105–111 GA model is much larger than an extreme premature human infant ( ~ 1.3–2.0 kg vs. 450–700 g) and more mature from a neurodevelopmental perspective, making it a less than ideal model for assessment of cardiovascular tolerance of the pumpless AV circuit and as a model of neurodevelopment. To address these discrepancies, in this study, we investigate EXTEND support of 90–95 day gestation lamb fetuses that more closely mimic the size and neurodevelopmental status^15–18^ of the human extreme premature infant.

We previously reported successful technical cannulation and short-term support (mean support length =120 h) of the extremely premature lamb (85–95 day GA). However, this study was, in retrospect, limited by cardiac failure due to increased afterload related to overzealous circuit flow restriction.^19^ Since this publication, the EXTEND project has evolved, as has our understanding of the physiology and care of fetal lambs in the EXTEND system. Here, we demonstrate that 90–95 day GA lambs can be maintained for up to 21 days with stable physiologic parameters.

## Methods

### Animals and surgery

All procedures were approved by the Institutional Animal Care and Use Committee (IACUC) of The Children’s Hospital of Philadelphia (IAC 19-00984).

Methods for anesthetic and surgical procedures have been previously described.^12,19^ Briefly, time-dated pregnant ewes were maintained under general anesthesia the fetus was delivered via a hysterotomy while preserving fetoplacental circulation. The two umbilical arteries and one umbilical vein were cannulated as previously described.^12^ The extracorporeal circuit consisted of minimal lengths of tubing and a low-resistance low-volume oxygenator (Quadrox-ID Pediatric Oxygenator, Maquet, Germany) primed with maternal blood. Fetuses were then transitioned to a sterile fluid environment where they were supported within the EXTEND system.^12^ At the end of the cannulation procedure, ewes were humanely euthanized.

### Experimental design

This study was conceived after we serendipitously encountered three animals with apparent in utero growth restriction (IUGR). The 3 animals had a mean gestational age of 107 days and a mean weight at cannulation of 0.76 kg (0.72–0.81 kg). While we initially considered that pregnancy was misdated in these animals due to physical characteristics closer to 90-95 GA fetuses, breeding dates were checked with the breeder and the gestational age was correct. The management of these smaller animals created a pilot study that enabled us to improve the management of extremely preterm animals on EXTEND. While the data from these animals (labeled IUGR) is included for interest, we did not perform detailed analysis and they are not included in the statistical comparisons of the experimental groups below.

Different study groups are summarized in Table 1. For the extremely preterm EXTEND group, a study duration of 10–14 days on EXTEND was chosen to span the duration of brain maturation up to the initiation of our previous lamb model of 105–111 days GA. This interval is also most relevant to the neurodevelopmental status of the 23–24 week gestation human. Animals were euthanized for analysis of end points within or at the end of this time interval independent of their potential for longer runs. The 10 animals included in this study met end of study protocol except 1 lamb that met end of protocol at 14 days but was maintained on EXTEND for long-term thrombogenicity study. This animal was not included in the brain analysis due the longer run on EXTEND making its brain more mature than other animals in this study.

**Table 1:** Study group design.

Group	GA	N	EXTEND duration	Brain MRI analysis	Brain histology analysis
Extremely preterm EXTEND*	90–95d	10	10–14 days	6	6
Extremely preterm Control	90–95d	5	N/A	0	0
Late gestation EXTEND**	105–112d	10	21–28 days	0	0
Late gestation Control	105–108d	4	N/A	3	3
Growth restricted EXTEND (IUGR)	105–108d	3	8–23 days	0	0

*Extremely preterm EXTEND group animals met end of study protocol between 10 and 14 days on EXTEND except 1 lamb that met end of protocol at 14 days but was maintained on EXTEND for long-term thrombogenicity study. Four lambs were excluded from study after premature death at 3, 6, 7 and 8 days on EXTEND from iatrogenic fluid overload, sudden cardiac arrest, and venous spasms respectively. Out of 10 study animals, 3 were excluded from brain MRI and histological analysis for unsuccessful brain perfusion and fixation, and the 21 day animal for gestational age difference.

**Late gestation EXTEND animals are historic cannulated lambs from our lab on the EXTEND system.

*GA* Gestational Age, *N* Number of lambs per group, *IUGR* Intrauterine growth restriction.

Four lambs were excluded from study having not met end of study protocol. One lamb died after 3 days on EXTEND from iatrogenic fluid overload having received a liter of intravenous fluid in less than an hour due to pump malfunction. The second lamb succumbed to sudden cardiac arrest with no preceding indications of cardiac failure after 6 days on EXTEND, prompting suspicion of air emboli. The final two lambs succumbed to sudden and irreversible venous spasms at 7 and 8 days, respectively. In both cases the un-sedated lambs likely induced venous spasm by kicking and stretching the short umbilical cord.

### Physiological monitoring during EXTEND support

Methods for maintaining lambs on EXTEND support including nutritional support, blood sampling and data acquisition are fully described in a previous report.^12^ Briefly, circuit blood flow, circuit pressures (systolic, diastolic, MAP), heart rate, umbilical artery and venous oxygen saturation, oxygenator sweep gas flow, oxygenator sweep and exhaust gas oxygen and carbon dioxide concentrations, oxygen delivery, oxygen consumption, oxygen extraction and incubator fluid temperature were measured in real time. Appropriate adjustments were made to nutritional support and oxygenator sweep gas parameters to maintain physiologic blood biochemistries and umbilical arterial and venous oxygen concentrations. Erythropoietin (400 U kg^−1^) was administered daily for hematocrit < 42% and adult packed red blood cells were transfused for hematocrit < 30%. An adjustable tubing clamp acting as a resistor to circuit blood flow was used to maintain estimated circuit blood flows around 325 ml/min/kg. For EXTEND animal groups, daily body weight was estimated based on initial weight at cannulation with a daily increase of 20 g/kg/day.^12^ Body weight at post-mortem was used to interpolate actual daily growth rate of EXTEND animals.

### Echocardiography

Cardiac ultrasound was performed daily to provide measurements of right and left ventricular cardiac outputs (RVCO, LVCO), combined cardiac output (CCO), ductus arteriosus flow (DA flow), ductus venosus pulsatility index (DVPI) and cardiac chamber dimensions. In addition, cardiac valve regurgitation was evaluated semi-qualitatively using an ordinal scale (no regurgitation, mild, moderate and severe regurgitation).

### Brain analysis

#### Cerebral MRI

Prior to euthanasia, lambs were systemically anticoagulated (2,000U Heparin), anesthetized with Etomidate (2 mg/kg) and humanely euthanized with potassium chloride (2mEq/kg). Via a median sternotomy, the brachiocephalic artery was cannulated and perfused via gravity (40cmH20) with ~500 mL of heparinized (10,000 IU) Plasmalyte; the superior vena cave (SVC) was sectioned to provide a fluid outflow path. When the SVC effluent was free of erythrocytes, the flush solution was replaced with 500 mL of 10% neutral buffered formalin (NBF). The brain was then removed, weighed and submerged in NBF. T1, T2, and T2* weighted sequences and diffusion tensor imaging (DTI) were obtained on a Bruker Neo Biospec 7 T machine (Bruker, Billerica, MA). Post-hoc MRI image analysis (Invicro, LLC, Boston, MA) was performed by a board-certified veterinary radiologist who was blinded to animal group. Out of 10 study animals, 3 were excluded from brain MRI and histological analysis for suboptimal brain fixation and 1 was excluded from brain analysis due to prolonged run on EXTEND making its brain more mature than other lambs included in the study.

Qualitative analysis included determination of the quality of the perfusion and fixation process (which led to exclusion of 3 brains), the presence/absence of intraventricular and white matter hemorrhages and/or lesions, and any gross anatomical abnormalities. For quantitative analysis, raw MRI images were digitally processed to remove signal noise from the container. Volumetric analysis was performed using VivoQuant (Scintica, Webster, TX) to calculate whole brain volumes and volumes for specific regions of interest including corpus callosum, gray matter volume, periventricular white matter layer 1, periventricular white matter layer 2 and total white matter volume which were normalized to total brain volume. All areas of interest were normalized to the total brain volume for each specimen. Transverse brain width was obtained at the level of the hippocampal commissure. Apparent diffusion coefficient,^20^ fractional anisotropy (FA), and diffusivity (axial and radial) were obtained using Diffusion Toolkit (TrackVis, Boston, MA). The regions of interest for this analysis were the same as those for the volumetric analysis listed above. For all analyses, the periventricular white matter was segmented manually and quality control was performed via peer review of the manually drawn regions of interest. Changes in ADC and FA in early brain maturation have been summarized to represent three major processes: fiber organization into fascicles, proliferation and maturation of glial cell bodies and intracellular compartments, and myelination.^21^ The use of MRI diffusion imaging has therefore emerged as a widely utilized method to identify white matter injury and monitor progression of white matter maturation.^22,23^

#### Histopathology

Histopathology analysis was performed by a board-certified veterinary pathologist at a GLP-certified laboratory (Horus Scientific Inc., Worcester, MA). Following MRI analysis, whole brains were sectioned according to Bolon et al. methodology^24^ and seven sections, from rostral to caudal, were analyzed for the assessment of the forebrain, midbrain, hindbrain, cerebellum, and brainstem. Brain tissue was paraffin embedded and stained with hematoxylin and eosin (H&E) stain, Fluoro-Jade B to study neuronal necrosis, Glial Fibrillary Acid Protein (GFAP) to study astrocytes and Ionized-calcium-Binding Adaptor molecule (IBA1) to study microglia. Morphologic changes were semi-quantitatively/qualitatively scored as follows: 0 (none), 1 (minimal), 2 (mild), 3 (moderate) and 4 (severe). Medullation was characterized by axonal sheath development and myelination. This was shown on histology by rarefaction and hypercellularity (H&E stain) with a concomitant decrease in positive astrocyte staining (GFAP stain) and increase in positive microglial staining (IBA-1 stain) as gestational age increases.

### Statistics

Hemodynamic (circuit flow and heart rate), oxygen and metabolic parameters were averaged over 24 h intervals. For analysis involving multiple measurements per animal such as hemodynamics, cardiac parameters, oxygenation parameters, fluid infusion rates, blood gases and biochemistries the unpaired T-test or Mann Whitney U-test depending on normal distribution (GraphPad Prism 9.3.1, Graph Pad Software, San Diego, California) were applied with significance accepted at *P* < 0.05 and data are presented as mean ± standard error of mean. Due to small sample size, single measurement parameters from the extremely preterm lamb group are presented descriptively with parameters from late gestation EXTEND animals as mean ± standard error of mean.Graphs display data points up to 21 days, however, group sizes in the extremely preterm group after 15 days on EXTEND was < 3 per group.

## Results

### Study group overview

General data per lamb has been summarized in Table 2. We included the data for the IUGR pilot group for interest and because of the prolonged survival of animal 1 which maintained stable parameters for 23 days despite a weight at cannulation of only 750 g encouraging us to pursue this study.

**Table 2:** Study group summary.

	Extremely preterm EXTEND group											Extremely preterm controls	Late gestation EXTEND	Late gestation controls	IUGR			
Number	1	2	3	4	5	6	7	8	9	10	MEAN ± SEM	MEAN ± SEM	MEAN ± SEM	MEAN ± SEM	1	2	3	MEAN ± SEM
**Sex (F/M)**	F	M	M	F	F	M	**M**	**F**	**M**	**F**					F	M	F	
**Age at cannulation (days)**	93	91	93	90	91	91	92	91	92	92	**91.6** ± **0.24**		**109.5** ± **0.67**		105	107	108	**106.7** ± **0.88**
**Weight at cannulation (kg)**	0.94	1.01	1.19	0.69	0.95	0.85	1.31	0.88	1.11	0.83	**0.98** ± **0.05**		**1.5** ± **0.087**		0.75	0.81	0.72	**0.8** ± **0.026**
**Size of arterial cannulas (Fr)**	10/10	10/10	10/10	10/10	10/10	10/10	10/10	10/10	10/10	10/10	**10/10**		**12/12**		10/12	12/12	12/12	
**Size of venous cannula (Fr)**	14	14	14	14	14	14	14	14	14	14	**14**		**14**		14	14	14	**14**
**Age at necropsy (days)**	104	112	107	104	107	105	102	104	104	102	**105.1** ± **0.94**	**90.8** ± **0.2**	**132.4** ± **1.19**	**105** ± **1.34**	128	115	119	**120.7** ± **3.8**
**Weight at necropsy (kg)**	1.86	1.97	1.86	1.02	1.49	1.34	1.47	1.51	1.76	1.47	**1.57** ± **0.09**	**0.72** ± **0.03**	**3.2** ± **0.09**	**1.34** ± **0.13**	1.762	1.210	1.180	**1.4** ± **0.19**
**Length of the run (days)**	11	21	14	14	16	14	10	13	12	10	**13.5** ± **1.04**		**22.9** ± **1.13**		23	8	11	**14** ± **4.58**
**Growth rate(g/kg/J)**	70.6	33.9	35.0	30.7	30.3	35.6	13.02	46.0	42.4	65.6	**40.3** ± 5.4*		**34.4** ± **0.005**		39.59	59.38	50.64	**49.9** ± **5.73**
**Brain weight at necropsy (g)**	27.5	28		22.2	32.7	26.1	22.6	26.1	30.8	23.3	**26.6** ± 1.2**	**16.2** ± **0.6**		**29.2** ± **0.6**				
**Brain to body weight**	14.7	14.2		21.7	22	19.6	15.3	17.3	17.5	15.9	**17.6** ± 1**	**22.7** ± **1.4**		**21.4** ± **1.2**				
**Brain to liver weight**	0.4	0.4		0.7	1.5	0.6	0.4	0.4	0.5	0.6	**0.48** ± 0.04 ***	**0.44** ± **0.02**		**0.49** ± **0.05**				
**Heart weight at necropsy (g)**	8.2	15.6	15.2	7.2	10.5	10.6	10.8	4.8	13.1	4.8	**10.1** ± **1.2**	**5.7** ± **0.5**		**10.2** ± **0.8**				
**Lung weight at necropsy (g)**	51.2	118.7	51.5	32.8	83.8	52.2	54.8	55.1	56.9	19.0	**57.6** ± **8.6**	**38.4** ± **1.6**		**63** ± **4.1**				
**Liver weight at necropsy (g)**	69.5	66.2	95.0	33.1	22.0	47.3	59.1	70.2	59.8	41.4	**56.4** ± **6.7**	**37** ± **1**		**62.6** ± **7.5**				
**Right kidney weight at necropsy (g)**	5.9	8.7	5.6	3.5	7.4	6.4	5.9	6.5	6.5	3.9	**6** ± **0.5**	**3.3** ± **0.2**		**5.9** ± **0.8**				
**Number of transfusions**	0	0	1	2	1	0	0	1	1	4	**1** ± **0.39**				0	0	2	**0.7** ± **0.67**

*Lamb 1 was excluded from growth rate mean because of excessive ascites.

**Lamb 3 is missing information for brain weight at necropsy

***Lamb 3 is missing information for brain weight at necropsy, Lamb 5 was excluded as outlier.

*IUGR* Intrauterine growth restriction, *SEM* Standard error of mean, *F* Female, *M* Male.

Mean + SEM values for each parameter are expressed in bold font for emphasis.

The extremely preterm group was composed of 10 animals with a mean gestational age of 91.6 days and a mean weight at cannulation of 0.98 kg (0.69–1.31 kg). There were no major technical complications during cannulation and all lambs were successfully transitioned to EXTEND. Nine animals were euthanized at the end of protocol (10–14 days). Animal #2 met end of protocol at 14 days but was maintained on EXTEND for 21 days with stable parameters for further for long-term thrombogenicity study.

### Hemodynamic and oxygenation parameters

Hemodynamic and oxygen parameters for all three IUGR and ten extremely preterm lambs are summarized and compared with late gestation EXTEND lambs in Supplemental Table 1.

#### Hemodynamic parameters

**Fig. 1:**
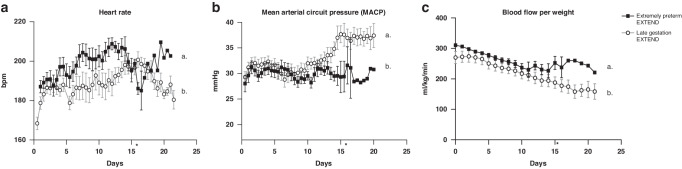
Hemodynamic parameters on EXTEND. **a** Heart rate. **b** Mean arterial circuit pressure (MACP). **c** Weight‐adjusted circuit flows. Data in (**a**–**c**) are represented in mean ±s.e.m. Statistical significance is expressed by different letters (a or b). Groups with the same letter show no statistical difference. *Size of extremely preterm EXTEND group after 16 days is *n* < 3.

Figure 1 shows the hemodynamic parameters of animals on our system, including heart rate, mean arterial circuit pressure (MACP) and weight-adjusted circuit blood flow. The extremely preterm group had an increased heart rate (Fig. 1a) and lower mean arterial circuit pressure (Fig. 1b – Supplementary Table 1) compared to older lambs on EXTEND (P < 0.0001) consistent with their earlier gestational age.^25,26^

Circuit blood flows per animal weight are shown in Fig. 1c. Late gestation lambs on EXTEND had been maintained with real weight adjusted circuit flows averaging 215.8 ml/kg/min without the need for an in-circuit resistor (Supplementary Table 1). For both IUGR and extremely preterm animals, presumably due to higher animal systemic vascular resistance and reduced autoregulatory capacity, circuit flows were supra-physiologic and were reduced via the addition of a tubing clamp resistor to maintain more physiologic umbilical flow. In the IUGR pilot group, estimated weight-corrected circuit flows were initially limited to ≤ 250 ml/kg/min based on our previous study, and based on a mean between different reference studies measuring normal umbilical flows in utero.^25,26^ We then progressively released the resistor to increase flows based on trial and error. Estimated circuit flows per weight were increased up to 400 ml/kg/min during this experience, however, daily cardiac ultrasound showed rapidly increasing cardiac output to supra-physiologic levels leading us to reduce circuit flows to avoid high output cardiac failure. Ultimately, limiting estimated circuit flow to ≤ 325 ml/kg/min (mean of 258.9 ml/kg/min real weight adjusted circuit flows) seemed to provide the best balance of systemic and circuit flow with normal cardiac outputs. Based on this pilot experience, the extremely preterm lambs were then maintained on higher circuit flow (mean=267.1 ml/kg/min) than our previous study (P < 0.0001). Over the course of the stay on EXTEND, there was a reduction in weight-corrected flow for all groups but flows remained in the physiologic range.

#### Oxygenation parameters

**Fig. 2:**
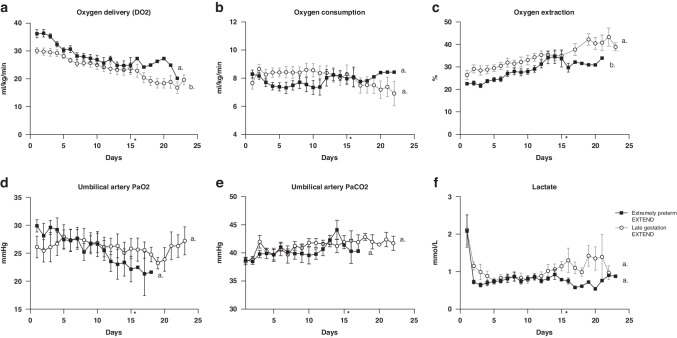
Oxygen Parameters on EXTEND. **a** Oxygen Delivery (DO2). **b** Oxygen consumption. **c** Oxygen extraction. **d** Umbilical artery partial pressure of oxygen (PaO2). **e** Umbilical artery partial pressure of carbon dioxide (PaCO2). **f** Lactate. Data in (**a**–**f**) are represented in mean ±s.e.m. Statistical significance is expressed by different letters (a or b). Groups with the same letter show no statistical difference. * Size of extremely pretermEXTEND group after 16 days is n < 3.

Oxygenation parameters for the extremely preterm and late gestation EXTEND animals are presented in Fig. 2.

The ability to measure umbilical artery (pre-oxygenator) and venous (post-oxygenator) oxygen saturations in real time enabled sustained and physiologic oxygen delivery to EXTEND animals (Fig. 2a). With a gradual decline in body-weight adjusted umbilical flow (Fig. 1c) there was a corresponding decrease in oxygen delivery over the course of the stay on EXTEND, however levels remained physiologic ( > 20 ml/min/kg) in both study groups (Fig. 2a). While general trend in body weight adjusted oxygen consumption was stable (Fig. 2b), there was a concomitant increase in oxygen extraction trend in both extremely preterm and late gestation groups to achieve physiologic oxygenation (Fig. 2c). Late-gestation EXTEND animals had similar oxygen consumption (*P* = 0.20) and lower oxygen delivery than extremely preterm lambs (P = 0.002). The lower oxygen delivery attributed to lower circuit flows resulted in higher oxygen extraction in the late gestation EXTEND group compared, to extremely preterm lambs (*P* < 0.001).

Adequate carbon dioxide exchange was similarly closely monitored via changes in sweep gas and was not different between groups (*P* = 0.84 – Fig. 2e).

PH (Supplementary Table 1) and Lactate (Fig. 2f) were used as an assessment of adequate gas exchange, and remained normal in all animal groups (*P* = 0.61 and *P* = 0.30 respectively).

Hemoglobin levels (Supplementary Table 1) in all groups progressively declined over time. Older lambs maintained a higher hemoglobin compared to the extremely preterm group(P = 0.03). Six lambs in the extremely preterm group needed at least one transfusion of adult blood due cumulative blood loss from blood sampling and umbilical cord bleeds while on EXTEND (Table 2). All transfusions given to the lambs in EXTEND were composed of 5–10 ml of adult packed red blood cells. Ewes were carefully selected to donate blood after agglutination testing was performed and no complications were observed during or after transfusions.

### Cardiac function

**Fig. 3:**
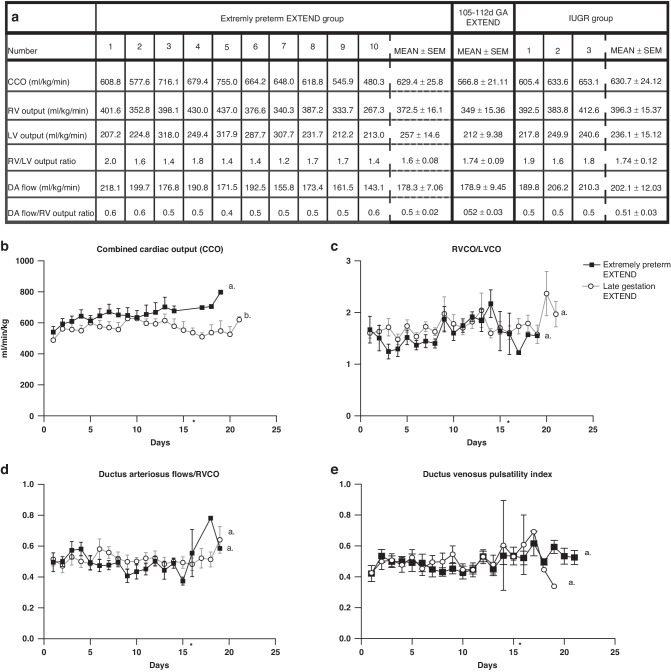
Echocardiographic parameters on EXTEND. **a** Echocardiographic parameters for each lamb. **b** Combined cardiac output. **c** Right ventricle cardiac output (RCVO) to left ventricle cardiac output (LVCO). **d** Ductus arteriosus (DA) flow to right ventricular output ratio. **e** Ductus venosus pulsatility index. Data in (**b**–**e**) are represented in mean ±s.e.m. Statistical significance is expressed by different letters (a or b). Groups with the same letter show no statistical difference. * Size of extremely preterm EXTEND group after 16 days is *n* < 3.

Echocardiographic data is summarized in Fig. 3a. Combined cardiac output (CCO) was higher for the extremely preterm group (*P* = 0.003) compared to older EXTEND animals (Fig. 3b) presumably due to their increased heart rate.^25,26^ Right to left ventricle cardiac output (Fig. 3c) and flow through the ductus arteriosus (Fig. 3d) were similar between study groups (*P* = 0.25 and *P* = 0.09 respectively) and similar to previously published data.^27,28^ Ductus venosus pulsatility index was monitored throughout the stay on EXTEND as abnormal DVPI has been associated with adverse pregnancy outcomes.^29–32^ In our study, the DVPI was stable and similar in both study groups (*P* = 0.96 – Fig. 3e). Cardiac valve regurgitation in extremely preterm lambs was assessed daily and ranked by severity of regurgitation. The aortic, pulmonary and mitral valves showed no regurgitation of moderate or severe degree and only occasional mild severity over time. No deterioration of the valve regurgitation was observed over time. The use of the resistor to limit circuit blood flow did not adversely impact cardiac afterload as evidenced by the lack of echocardiographic evidence of valvular insufficiency. The tricuspid valve shows mostly mild regurgitation over time with 1 episode of moderate regurgitation on 1 day. Data summarizing valve regurgitation is represented in Supplemental Fig. 2. Cardiac contractility was subjectively qualitatively assessed by the ultrasonographer as good throughout without any significant dysfunction and chamber size could be used as an indicator of general volume status, allowing adjustments in fluid administration.

### Brain analysis

#### Cerebral MRI

**Fig. 4:**
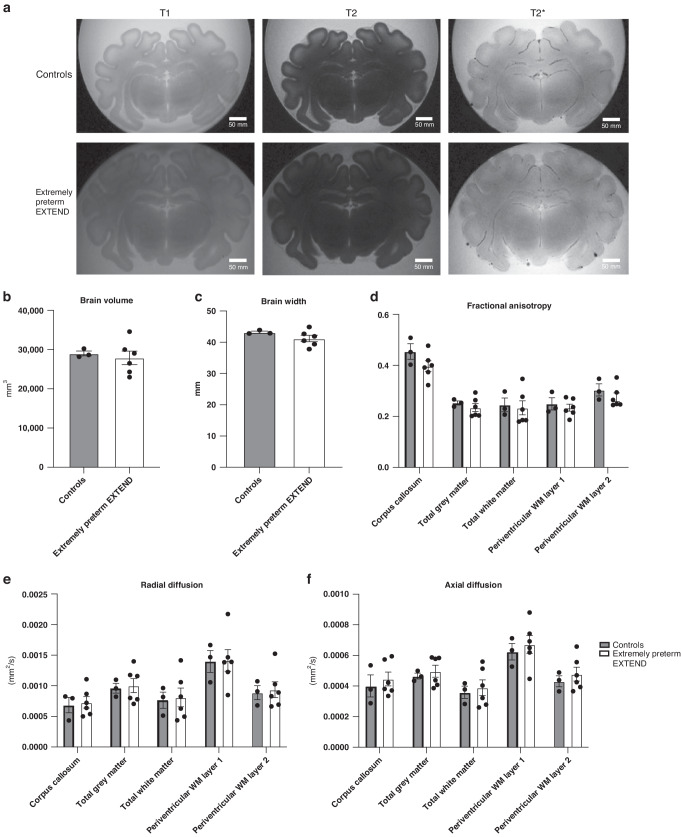
Brain MRI analysis. **a** Qualitative analysis: Control and extremely preterm EXTEND group mid-brain MRI in T1, T2 and T2* mode. **b** Total brain volume. **c** Transverse brain width. **d** Fractional anisotropy. **e** Radial diffusion per brain region. **f** Axial diffusion per brain region. WM: White Matter. Data in (**b**–**f**) are represented in mean ±s.e.m.

Qualitative analysis of the brains did not show any signs of intraparenchymal hemorrhage or infarction (Fig. 4a).

##### Brain growth.

Average absolute brain volumes (Fig. 4b) were 29024.4 vs 27906.9 mm^3^ and brain widths (Fig. 4c) were 43.2 vs 41.2 mm, in gestationally equivalent control lambs versus extremely preterm EXTEND animals respectively and the differences were distributed equally among brain regions with apparent preservation of volumetric ratios (Fig. 4c). Different volumetric characteristics per brain region are summarized in Supplemental Fig. 1g.

##### Brain maturation.

Diffusion analysis is shown in Fig. 4d–f. Fractional anisotropy, radial diffusivity and axial diffusivity appears comparable between groups.

#### Histopathology

The macroscopic analysis of brain slabs did not reveal any brain bleeds or clot formation. Macroscopic observations of histological slides show small red/brown discoloration/accumulation on the meningeal surface in three extremely preterm EXTEND animals and one late gestation control. The most common macroscopic observation was the presence of minimal hemorrhage within the meninges and/or neuropil regardless of the animal group. These findings were attributed as acute peri-mortem injuries due to the minimal magnitude of the morphological change and lack of evidence of chronicity on histopathology such as erythrophagocytosis or hemosiderosis. Microscopic analysis showed evidence of progressive medullation (axonal sheath development and myelination) of the radiating cerebrocortical white matter tracts in both study groups. In addition, the presence of progressive medullation was interpreted to be consistent with end of the study gestational ages of the extremely preterm lambs. This, in combination with the MRI diffusion analysis, supports qualitatively similar maturation and myelination in our experimental group. Different stains are represented in Supplemental Fig. 3.

### Growth and metabolism

Animals visually matured while in EXTEND in all groups, with development of thicker skin, wool, and pigmentation as well as stable growth rate compared to older lambs on EXTEND Supplementary Figure 4a). Animal brain to body weight ratio (Supplementary Figures 4b, 21.4 vs 17.6) appeared lower in extremely preterm animals at time of necropsy compared to age-matched in utero controls whereas the brain to liver weight ratio (Supplementary Fig. 4c, 0.49 vs 0.48) was similar. Absolute brain weight values in extremely preterm EXTEND animals were larger than extremely preterm controls (26.6 g vs. 16.2 g) consistent with some growth but were on average smaller than late gestation controls (Tables 2, 29.19 g vs 26.57 g). Gross examination of the brains revealed obvious maturational changes with increased sulcation relative to extremely preterm controls (Supplementary Figure 1a–f).

Gross examination of other organs showed that the heart (10.2 g vs 10.1 g – difference 1.1%), lung (63 g vs 57.59 g – difference 8.56%), liver (62.55 g vs. 56.35 g– difference 9.91%) and right kidney (5.9 g vs. 6.03 g – difference 2.2%) weight were similar between the extremely preterm group and age-matched in utero controls, suggesting no cardiac hypertrophy or lung hypo- or hyperplasia. When compared to organ weights of extremely preterm controls (Table 1) all show clear organ growth. Cardiac cavities were measured with cardiac ultrasound and showed growth over time with no visible dilation. Extremeley preterm lambs’ final measurement is equivalent to the older lambs’ first measurement (Supplementary Fig. 5).

Other metabolic parameters used as markers for organ function and nutritional status were measured throughout their stay on EXTEND (Supplementary Fig. 6a). Liver function tests showed that total bilirubin was increased in all experimental groups compared to controls. However, other hepatic enzymes including AST, ALT, and alkaline phosphatase were similar to previous published literature.^12^ Cholesterol showed a rapid decrease over time justifying an increase in lipid administration.

Because of the very small size of the lambs at 90–95 days of GA, decreased intravenous fluids (*P* < 0.001) were administered to the extremely preterm lambs to avoid increased iatrogenic third-spacing (Supplementary Fig. 6b).

## Discussion

The EXTEND system has previously been shown to support premature lambs for up to 4 weeks in a fetal physiologic state with the goal of ultimately providing a platform for support of preterm infants and those with other conditions who would benefit from maintenance of the fetal state. Previous work in this model has shown that 105–110 days GA animals, corresponding in lung maturity to the 22–25-week human fetus, sustain normal hemodynamics and multi-organ growth and development in the EXTEND system.^12^ Because the study was based on lung development timelines, 105 days of GA lambs were significantly larger than their human counterparts. This facilitated early studies as larger lambs are easier to surgically manipulate and have a large umbilical cord facilitating cannulation in the early evolution of EXTEND.

With regard to brain development, human 23–32 week GA infants undergo important sulcation, myelination, and an increase in cerebral and cerebellar volumes. In addition, synaptic density increases, thalamocortical projections develop, and neurons migrate to the cortex.^33^ Preterm infants born at this age have a predisposition for cerebral white matter injury^33,34^ as well as germinal matrix and intraventricular hemorrhage (GMH-IVH). In comparison, the fetal sheep brain development progresses more rapidly compared to the human fetal brain. Studies show that 95 day GA lamb brain corresponds to the 24 to 28 week GA human brain in terms of the completion of neurogenesis, the onset of cerebral sulcation, and the detection of the cortical component of the auditory and somatosensory evoked potentials.^15–18^ Furthermore, oligodendrocyte progenitors, thought to be responsible for diffuse myelination disturbances when injured, are seen in human fetal brains up to 30 weeks of GA^35^ whereas, in their ovine counterparts, evolution of progenitors to mature oligodendrocytes starts at around 90 days of GA and is largely achieved by 105–110 days of GA.^34,36^ Therefore, the immaturity of the 90–105 day GA fetal periventricular white matter supports this gestational age as the optimal developmental window to study white-matter injury in the lamb.^23,34,36^ In contrast, there is little evidence supporting the extremely preterm lamb model as a model of GMH/IVH, likely because even at 90 days, the germinal matrix is relatively mature. It is important to note that challenges of supporting premature lambs ex-utero have led researchers to focus on studying GMH/IVH in fetal lambs instead, so the conditions causing GMH/IVH in human premature infants cannot be easily replicated (i.e. the transitional circulation and ventilation). Attempts to induce GMH/IVH in extremely preterm lambs through hypoxia, hypovolemia, hypervolemia or brief umbilical embolization (up to 20 min) were unsuccessful.^37^ Reynolds et al.^38^ reported germinal matrix hemorrhage in 30% of exteriorized lambs aged 58 to 85 days of gestation (average gestational age 73 days) when exposed to arterial and venous hypertension with asphyxia. Thus, even at this earlier gestation time-point, it required extreme fluctuations in vascular pressure as well as asphyxia to induce GMH/IVH.^39,40^ The previously published data therefore, negate the potential usefulness of the extremely preterm lamb as a model for GMH/IVH, particularly in a physiologically stable system like EXTEND. Thus, our observation of no GMH/IVH would be anticipated in this study, and is not relevant to the extreme premature human infant with regard to risk of GMH/IVH. Unfortunately, experimental assessment of this important clinical risk, cannot be relevantly accomplished in the normal fetal lamb model.

Several changes in the physiologic management of animals on EXTEND allowed us to maintain these extremely preterm lambs on circuit. The EXTEND system is made of a short pumpless circuit connected to small volume near-zero-resistance oxygenator to minimize cardiac afterload and closely mimic the normal fetal/placental circulation. 105-111 days gestation lambs have the vasoactive maturity to autoregulate circuit flows to achieve an optimal balance of circuit and systemic flow. This allows physiologic cardiac function while on EXTEND. However, younger lambs have higher systemic vascular resistance and less autoregulatory capacity leading to preferential shunting of cardiac output through the circuit. This ultimately causes hypoxemia and resulting lactic acidosis. The lambs attempt to combat this by increasing cardiac output, which further increases circuit flow but cannot meet systemic perfusion demand, ultimately leading to cardiac failure.^41,42^ Based on the results of our previous small animal study,^19^ and on trial and error in our pilot study of IUGR animals, we set maximal circuit flow in this study at around 325 ml/kg/min acheiving a sustainable balance of cardiac output and circuit flows, and allowing survival of extremely preterm lambs on circuit for up to three weeks and an average of 13.5 days with normal cardiac growth without cardiac failure

This physiologic stability on EXTEND then allowed us to assess neurodevelopment in the 90–95 day GA lamb over the duration of the period when the brain is at high risk of white matter injury. We performed MRI imaging and detailed analysis of the brains of experimental animals in comparison with end-of-study matched controls. Notably, no ischemic or white matter injury was seen in any of the experimental animals. The brains in the extremely preterm group were smaller in size compared to age-matched control animals on brain weight and volumetric analysis, and the brain to body weight ratios were approximately 18% smaller. Reasons for this discrepancy are likely related to body wall edema and effusions, observed in some of the lambs, that accumulated near the end of the run. This explanation is supported by the 0.9% difference in brain to liver ratios as liver and other organ edema was minimal on necropsy. It is also possible that brain/organ growth could be impacted by our inability to exactly replicate normal fetal lamb nutrition with human total parenteral nutrition formulations, and specifically our lack of knowledge of lamb lipid requirements for normal brain development. Nevertheless, the brains did grow when compared to the extremely preterm controls and sulcation, as assessed by gross examination suggested maturation of the brains on EXTEND. Further analysis of the brain through gross, histopathologic and MRI analysis showed no GMH/IVH in extremely preterm EXTEND and control animals. Myelination was also qualitatively assessed through histopathological analysis showing no apparent inhibition or abnormality of the myelination process observed in the extremely preterm EXTEND animals compared to age-matched controls in agreement with the MRI diffusion analysis. However, it is important to note that because of the sample size and variability inherent in the sheep model, this study is underpowered to detect even major differences in brain growth or development, and that many of these parameters are qualitative.

Other areas where improvements were made included tighter oxygenation management and blood-loss management. Lambs in the EXTEND system have exposure to adult hemoglobin from priming and transfusions, which shifts the oxygen-hemoglobin dissociation curve to the right.^43,44^ Thus, PaO2 was slightly higher compared to prior studies to maintain the same oxygen saturation.^12,19,43^ In addition, we limited adult donor blood transfusions by making several changes to limit the amount of adult blood administered. These measures included decreasing the size of the circuit, decreasing the frequency of blood draws for labs, and changes to the biobag to prevent animal injury in the bag. With these measures, only 6 of 10 animals in the extremely preterm group required one or more maternal blood transfusion.

While this data shows that the extend system can successfully maintain extremely preterm lambs on circuit, it also highlights the importance of close echocardiographic and physiological monitoring of lambs on circuit that will be crucial to the successful translation of this technology.

## Limitations

Firstly, the sample size in our current study is small due to the resource-intensive nature of maintaining fetal lambs on EXTEND. Secondly, although data in the lamb model is informative, the circulatory reaction and autoregulatory capacity of the human fetus to a modified EXTEND device remains to be seen. Particular attention to the circuit flow management of human patients will need to be applied despite the knowledge acquired in this study. Third, the extremely preterm lamb model is not predictive of human GMH/IVH of prematurity.^39^ Finally, the examination of myelination through luxol fast blue staining could not be achieved due to preemptive gadolinium treatment. Furthermore, conclusions based on histologic and MRI analysis of normal brain development are limited to the resolution of the methodology and can only be interpreted as a qualitative assessment.

## Conclusion

This study demonstrates the prolonged support of extremely preterm lambs (91–93 day GA) on EXTEND with improved management. The lambs demonstrate normal oxygen delivery and metabolism with controlled circuit flows for up to 21 days. No cardiac failure was observed and extremely preterm lambs showed no additional complications compared to older lambs on EXTEND. In addition, cannulated lambs have no signs of brain injury on MRI or on histopathological findings. This new data provides new insights into fetal physiology on EXTEND.

## Supplementary information

Supplemental figures_small animal-R3 paper

## Data Availability

The datasets generated during and/or analyzed during the current study are not publicly available due to an ongoing FDA submission but are available from the corresponding author on reasonable request.
