# Supplementary material for: Assessment of extremely premature lambs supported by the Extrauterine Environment for Neonatal Development (EXTEND)
Source: Pediatr Res. 2024 Jun 4;96(7):1616–25. doi: 10.1038/s41390-024-03287-0 (PMC11772227; doi:10.1038/s41390-024-03287-0)
Supplement: Supplementary file 1 — Supplemental figures_small animal-R3 paper [file 41390_2024_3287_MOESM1_ESM.pdf]

	Extremely preterm EXTEND group											Late gestation EXTEND	IUGR			
**Number**	**1**	**2**	**3**	**4**	**5**	**6**	**7**	**8**	**9**	**10**	**MEAN ± SEM**	**MEAN ± SEM**	**1**	**2**	**3**	**MEAN ± SEM**
**Hemoglobin (g/dl)**	9.7	8.0	7.4	8.4	8.4	9.4	9.2	9.5	9.1	7.1	**8.6 ± 0.3**	**11 ± 0.5**	8.9	9.6	6.6	**8.4 ± 0.23**
**Heart rate (bpm)**	198.0	189.0	189.1	198.9	200.1	198.3	221.3	213.8	209.5	197.6	**201.5 ± 3.3**	**186.9 ± 3.53**	188.1	186.3	196.6	**190.3 ± 3.17**
**Circuit flow/weight (ml/kg/min)**	236.6	282.0	275.4	280.6	271.9	271.8	286.5	258.5	243.7	241.5	**264.9 ± 5.8**	**217.6 ± 17.6**	249.9	255.6	265.6	**257.1 ± 4.6**
**Pre-systolic pressure (mmHg)**	38.6	43.5	46.0	53.8	48.1	57.9	52.3	47.0	46.2	48.1	**48.2 ± 1.7**	**50.3 ± 1.22**	48.6	42.3	49.2	**46.7 ± 2.22**
**Pre-diastolic pressure (mmHg)**	21.1	21.0	18.5	21.9	24.2	21.9	22.4	22.6	20.5	19.3	**21.3 ± 0.5**	**27.6 ± 0.95**	26.8	22.5	24.4	**24.6 ± 1.26**
**Pre-MAP (mmHg)**	25.4	25.7	27.6	32.5	32.2	34.0	32.4	30.8	29.1	28.9	**29.9 ± 0.9**	**33.9 ± 1.01**	33.1	26.9	31.3	**30.4 ± 1.85**
**Post membrane 02 saturation (%)**	70.3	70.0	76.1	68.8	74.0	65.1	72.0	71.8	70.4	70.2	**70.9 ± 0.9**	**71.9 ± 1.36**	78.8	75.7	79.0	**77.8 ± 1.06**
**Total O2 delivery (ml/kg/min)**	28.7	29.1	29.4	29.4	30.3	29.9	33.9	30.3	27.4	24.7	**29.3± 0.7**	**24 ± 0.1.32**	30.2	31.5	24.2	**28.8 ± 2.05**
**Total O2 consumption (ml/kg/min)**	6.6	7.1	8.4	8.3	8.6	7.9	8.0	8.3	8.1	5.7	**7.7 ± 0.3**	**8 ± 0.43**	7.8	7.2	6.4	**7.2 ± 0.39**
**Total O2 extraction (%)**	24.0	24.4	29.5	29.0	28.6	27.0	23.7	28.2	31.7	24.8	**27.1 ± 0.9**	**35.9 ± 1.92**	27.5	24.1	30.1	**27.2 ± 1.74**
**Umbilical artery PaCo2 (mmHg)**	39.2	40.5	41.2	39.7	41.5	39.1	41.9	40.2	41.1	37.5	**40.2 ± 0.4**	**41.3 ± 1.8**	40.1	41.3	40.1	**40.5 ± 0.41**
**Umbilical artery PaO2 (mmHg)**	31.5	24.9	20.6	34.7	23.7	23.1	23.3	31.2	23.9	29.6	**26.7 ± 1.5**	**22.6 ± 1.1**	31.1	31.5	37.9	**33.5 ± 2.19**
**Umbilical artery O2 saturation (%)**	58.8	59.2	59.6	59.3	59.9	58.6	60.0	58.0	58.7	59.4	**59.1 ± 0.2**	**53.4 ± 1.72**	60.9	62.3	59.3	**60.8 ± 0.86**
**Umbilical artery PH**	7.4	7.4	7.4	7.4	7.4	7.4	7.4	7.4	7.4	7.4	**7.4 ± 0**	**7.4 ± 0.4**	7.4	7.4	7.4	**7.4 ± 0.01**
**Plasma lactate (mmol/l)**	0.8	0.8	0.7	1.0	0.9	0.7	1.3	0.7	0.8	1.5	**0.9 ± 0.1**	**1 ± 0.1**	1.0	0.8	0.9	**0.9 ± 0.27**

Supplemental Table 1. Hemodynamic and oxygen parameters of IUGR and Extremely preterm EXTEND group.
MAP : Mean arterial pressure – IUGR : Intrauterine growth restriction.

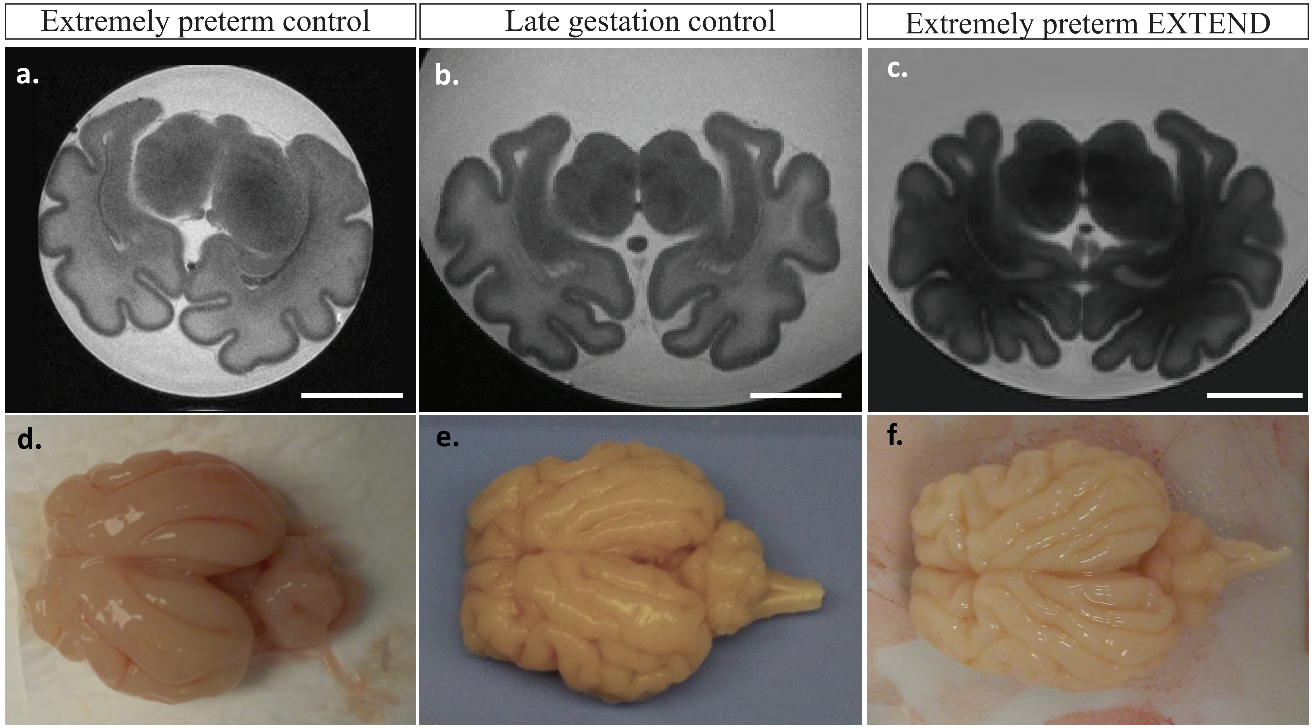

g.	Extremely preterm EXTEND group						Late gestation controls		
**Number**	**3**	**5**	**6**	**7**	**8**	**9**	**1**	**2**	**3**
**Age at cannulation (days)**	93	91	91	92	91	92	107	105	105
**Length of the run (days)**	14	16	14	10	13	12			
**Transverse Brain Width (mm)**	44.9	42.7	39.4	40.5	37.8	41.9	43.9	42.9	42.8
**Corpus Callosum**	0.001	0.001	0.002	0.002	0.001	0.003	0.002	0.001	0.002
**Periventricular White Matter Layer 1**	0.007	0.007	0.007	0.010	0.007	0.014	0.007	0.006	0.007
**Periventricular White Matter Layer 2**	0.008	0.008	0.008	0.010	0.008	0.014	0.008	0.007	0.008
**Total Periventricular White Matter**	0.015	0.014	0.015	0.020	0.015	0.028	0.015	0.013	0.016
**Total White Matter Volume**	0.405	0.426	0.408	0.308	0.333	0.435	0.342	0.350	0.430
**Total Gray Matter Volume**	0.596	0.574	0.592	0.692	0.667	0.565	0.658	0.650	0.571

Supplemental Figure 1 Structural development and volumetric characteristics of MRI analysis.
a. Brain MRI of extremely preterm control (91 days of gestation). – **b.** Brain MRI of Late gestation control (105 days of gestation). – **c.** Brain MRI of extremely preterm EXTEND (105 days of GA). a-c scale bar represents 10mm – **d.** Brain photo of extremely preterm control (91 days of gestation). – **e.** Brain photo of Late gestation control (105 days of gestation). – **f.** Brain photo of extremely preterm EXTEND (105 days of GA). – **g**. MRI Volumetric analysis of extremely preterm EXTEND group and Late gestation controls. All measures except transverse brain width are normalized on animal brain volume

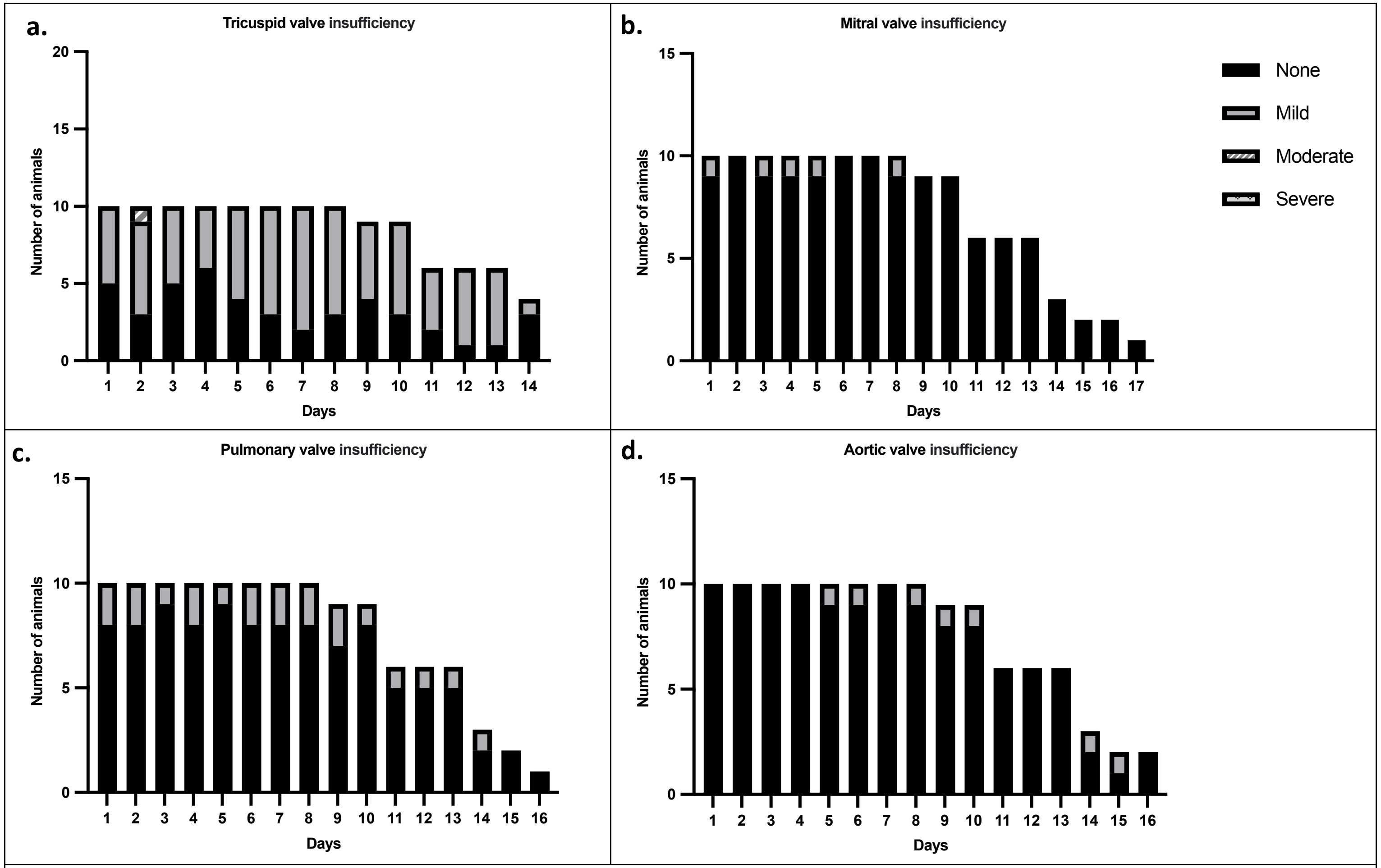

Supplemental Figure 2. Echocardiographic valve regurgitation in extremely preterm lambs on EXTEND
a. Tricuspid valve regurgitation. – **b**. Mitral valve regurgitation. – **c.** Pulmonary valve regurgitation. – **d.** Aortic valve regurgitation.

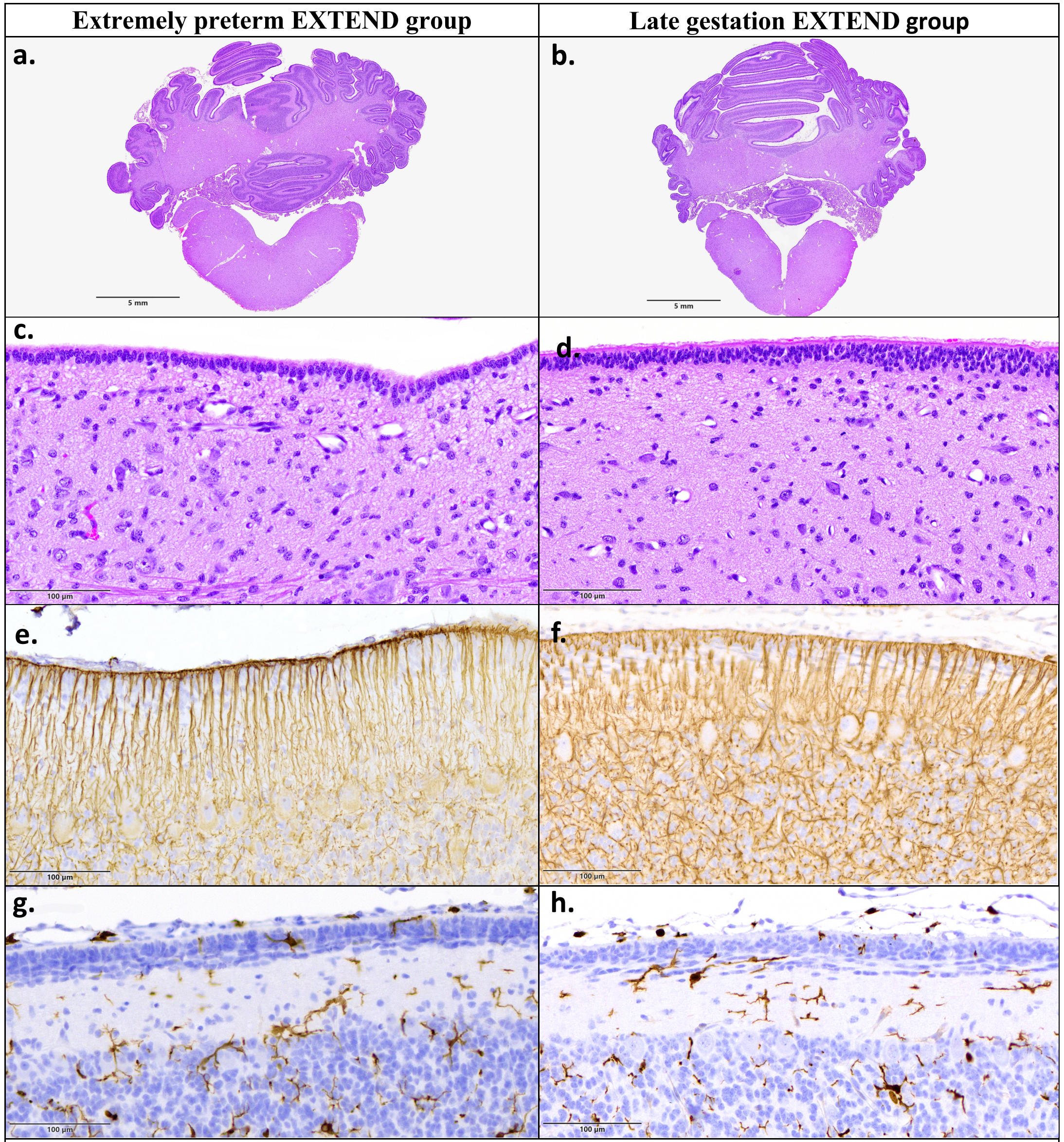

<u>Supplemental Figure 3.</u> Brain Histopathology

a. Cerebellum of extremely preterm EXTEND group stained with H&E. – **b.** Cerebellum of control lamb stained with H&E. – **c.** Midbrain of extremely preterm EXTEND group stained with H&E. – **d.** Midbrain of control lamb stained with H&E. – **e.** Cerebellum of extremely preterm EXTEND group stained with GFAP. – **f.** Cerebellum of control lamb stained with GFAP. – **g.** Cerebellum of extremely preterm EXTEND group stained with IBA1. – **h.** Cerebellum of control lamb stained with IBA1.

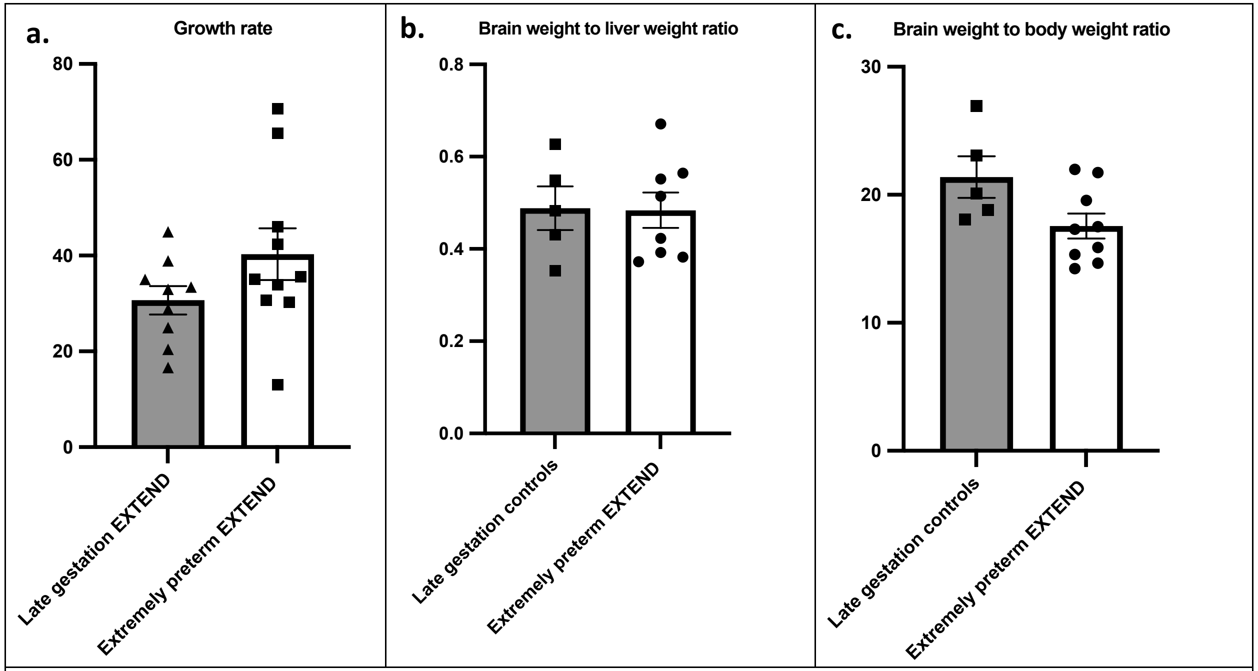

Supplemental Figure 4. Growth rate and Growth index.

a. Growth rate on EXTEND. – **b**. Brain to liver weight ratio (LBR) between Extremely preterm EXTEND group and late gestation control. – **c.** Brain to body weight ratio (BBR) between extremely preterm EXTEND group and late gestation control. Lamb 1 was excluded in a. and c. because of excessive ascites. Data in A, B and C are represented in mean ±s.e.m.

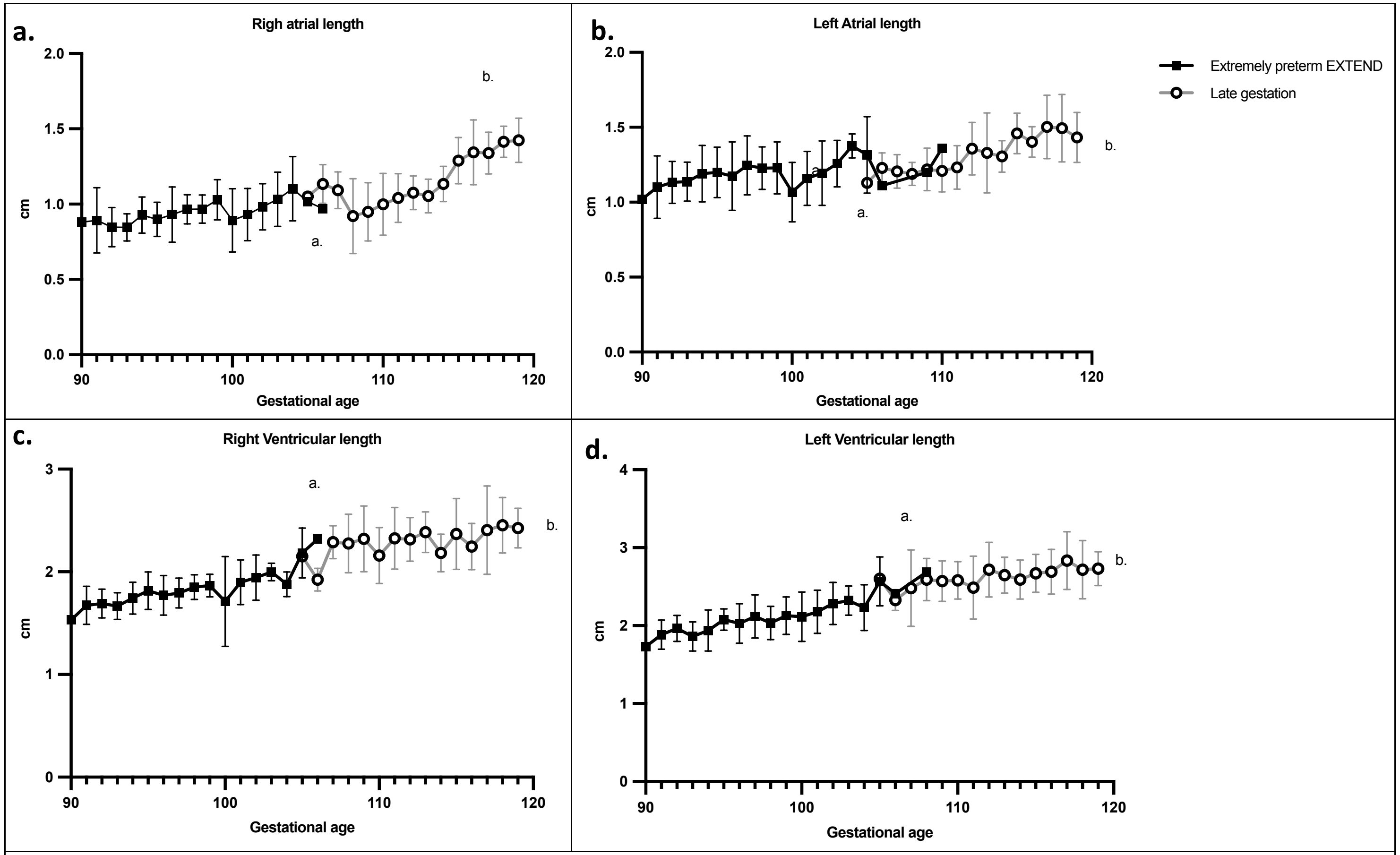

<u>Supplemental Figure 5</u> Echocardiographic parameters of cardiac cavity size.
a. Right atrial length. – **b.** Left atrial length. – **c.** Right ventricular length. – **d.** Left ventricular length. Data in b, c, and d are represented in mean ±s.e.m. Statistical significance is expressed by different letters (a, b or c). Groups with the same letter show no statistical difference.

a.	Extremely preterm EXTEND group		Late gestation EXTEND*		Extremely preterm Control	Late gestation controls	IUGR group	
**Days or run**	**Days 0-7 (n=13)**	**Days 7-15 (n=11)**	**Days 0-7 (n=5)**	**Days 7-14 (n=5)**	**MEAN ± SEM (n=4)**	**MEAN ± SEM (n=4)**	**Days 0-7 (n=3)**	**Days 7-11 (n=3)**
**Total Protein (g/dl)**	3.1 ± 0.5	2.6 ± 0.2	3.5	3.3	2,7 ± 0,1	3.3 ± 0.02	3 ± 0.05	2.63 ± 0.4
**AST (UI^{-1})**	36.1 ± 7.1	29.3 ± 1.6	35.00	28.00	25,5 ± 1,9	24.5 ± 1.5	23.5 ± 3.5	33.75 ± 2.5
**ALT (UI^{-1})**	10.1 ± 1.6	9.1 ± 1.3	5.00	3.00	6,7 ± 0,6	14.1 ± 2.4	6 ± 3	5 ± 2
**Alk Phos (UI^{-1})**	67.6 ± 15.8	77.3 ± 23.6	126.00	93.00	115 ± 18,1	132.9 ± 5	80 ± 6	77.3± 22.3
**Total Bili (mg/dl)**	2.2 ± 1.08	3.8 ± 0.4	1.1	2.2	0,9 ± 0,2	0.7 ± 0.1	1.5 ± 0,1	2 ± 0.5
**BUN (mg/dl)**	22.7 ± 2.2	26.4 ± 1.7	23.00	23.00	21,7 ± 2,1	24.5 ± 0.2	23.6 ± 3.1	30.8 ± 2.3
**Creatinine (mg/dl)**	0.5 ± 0.1	0.5 ± 0.04	0.71	0.77	0,6 ± 0,1	1.3 ± 0.4	0.3 ± 0.03	0.4 ± 0.05
**BUN/CREA**	49.7 ± 4	53.7 ± 3.2	32.39	29.87	41,2 ± 4,8	23.9 ± 4.1	86 ± 8	80.4 ± 6.1
**Phos (mEq/L)**	5.93 ± 1.2	5.1 ± 0.7	7.3	4	6,5 ± 0,17	6.6 ± 0.4	5.9 ± 0.8	3.4 ± 0.5
**Glucose (mg/dl)**	27.3 ± 1.8	27.4 ± 2.6	26.00	27.00	39,3 ± 10,7	39.8 ± 3	27.1 ± 2.7	25 ± 0.5
**Total Ca (mg/dl)**	11.8 ± 0.9	12.5 ± 0.8	10.8	12.1	12,2 ± 0,4	12.9 ± 0.1	9.9 ± 0.4	12.2 ± 0.8
**Na (mEq/L)**	139.8 ± 0.9	140.9 ± 1.1	139.00	139.00	137,2 ± 0,9	139.3 ± 0.9	140 ± 0.6	139.1 ± 0.7
**K (mEq/L)**	4.1 ± 0.1	4.1 ± 0.09	4.1	4	4,2 ± 0,4	4.3 ± 0.1	4.1 ± 0.1	4.2 ± 0.1
**Cl (mEq/L)**	105.5 ± 1.2	105.8 ± 1.1	104.00	104.00	105,2 ± 1,7	103.3 ± 1.1	104.9 ± 0.9	104.6 ± 1.1
**Cholesterol (mEq/L)**	30.8 ± 4.8	22.8 ± 4.3	31.00	23.3	33,6 ± 1,5	29.8 ± 1.2	49.5 ± 10.5	19 ± 3
**Mg (mg/dl)**	1.97 ± 0,2	2.16 ± 0,1	.	.	2,2 ± 0,2	2.1 ± 0.1	.	.
**Fluid intake (ml/kg/hr)**	16.3 ± 1.3	13.5 ± 0.6	17.4 ± 2.1	16.1 ± 1.4			18.4 ± 1.3	17.2 ± 0.4

b.

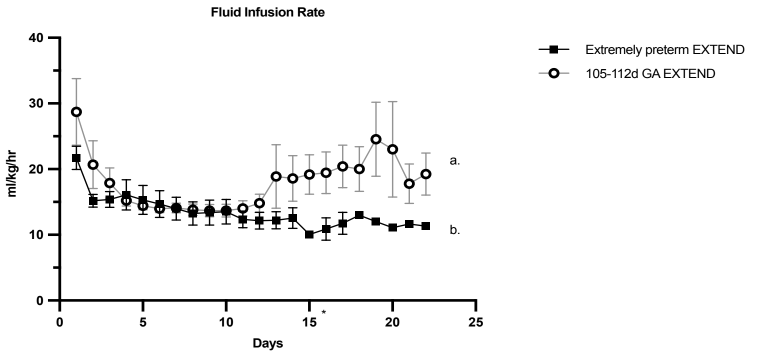

Supplemental Figure 6. Metabolic and Fluid parameters

a. Metabolic and fluid parameters summarized. *Data for the late gestation EXTEND group was taken from previously published data (Partridge et al. (2017), Nature comms) – **b.** Fluid intake. Extremely preterm EXTEND group show a significantly lower infusion rate than late gestation group (P=0.04). Statistical significance is expressed by different letters (a and b). Groups with the same letter show no statistical difference. * Size of extremely preterm EXTEND group after 16 days is n < 3.
